# Sarcopenia and sarcopenic obesity among older adults in the nordic countries: a scoping review

**DOI:** 10.1186/s12877-024-04970-x

**Published:** 2024-05-13

**Authors:** Fereshteh Baygi, Sussi Friis Buhl, Trine Thilsing, Jens Søndergaard, Jesper Bo Nielsen

**Affiliations:** Research Unit of General Practice, Department of Public Health, University of Southern Denmark, Odense, Denmark

**Keywords:** Age, Sarcopenia, Sarcopenic obesity, Prevalence, Nordic countries

## Abstract

**Background:**

Sarcopenia and sarcopenic obesity (SO) are age-related syndromes that may compromise physical and mental health among older adults. The Nordic countries differ from other regions on prevalence of disease, life-style behavior, and life expectancy, which may impact prevalence of sarcopenia and SO. Therefore, the aim of this study is to review the available evidence and gaps within this field in the Nordic countries.

**Methods:**

PubMed, Embase, and Web of science (WOS) were searched up to February 2023. In addition, grey literature and reference lists of included studies were searched. Two independent researcher assessed papers and extracted data.

**Results:**

Thirty-three studies out of 6,363 searched studies were included in this scoping review. Overall prevalence of sarcopenia varied from 0.9 to 58.5%. A wide prevalence range was still present for community-dwelling older adults when definition criteria and setting were considered. The prevalence of SO ranged from 4 to 11%, according to the only study on this field. Based on the included studies, potential risk factors for sarcopenia include malnutrition, low physical activity, specific diseases (e.g., diabetes), inflammation, polypharmacy, and aging, whereas increased levels of physical activity and improved dietary intake may reduce the risk of sarcopenia. The few available interventions for sarcopenia were mainly focused on resistance training with/without nutritional supplements (e.g., protein, vitamin D).

**Conclusion:**

The findings of our study revealed inadequate research on SO but an increasing trend in the number of studies on sarcopenia. However, most of the included studies had descriptive cross-sectional design, small sample size, and applied different diagnostic criteria. Therefore, larger well-designed cohort studies that adhere to uniform recent guidelines are required to capture a full picture of these two age-related medical conditions in Nordic countries, and plan for prevention/treatment accordingly.

**Supplementary Information:**

The online version contains supplementary material available at 10.1186/s12877-024-04970-x.

## Background

The number of older adults with age-related disorders is expected to increase worldwide [1, 2]. Sarcopenia and sarcopenic obesity (SO) are both age-related syndromes that may compromise the physical and mental health of older adults and increase their need for health care services in old age [3, 4], and this may challenge the sustainability of health care systems economically and by shortage of health care personnel [5].

Sarcopenia is characterized by low muscle mass in combination with low muscle strength [4]. SO is characterized by the co-existence of obesity (excessive adipose tissue) and sarcopenia [3]. Sarcopenia and SO are both associated with physical disability, risk of falls, morbidity, reduced quality of life and early mortality [4, 6–9]. In SO the consequences of sarcopenia and obesity are combined and maximized [4, 6–8].

Etiology of sarcopenia and SO is multifactorial and closely linked to multimorbidity [3, 7–10]. Nevertheless, lifestyle and behavioral components particularly diet and physical activity, are important interrelated factors that potentially can be modified. Physical inactivity and sedentary behavior may accelerate age-related loss of muscle mass, reduce energy expenditure, and increase risk of obesity [3, 11]. In addition, weight cycling (the fluctuations in weight following dieting and regain) and an unbalanced diet (particularly inadequate protein intake) may accelerate loss of muscle mass and increase severity of sarcopenia and SO in older adults [3, 12]. International guideline for the treatment of sarcopenia emphasizes the importance of resistance training potentially in combination with nutritional supplementation to improve muscle mass and physical function [13]. Similar therapeutic approach is suggested for treatment of SO [14]. However, more research is needed to confirm optimal treatment of SO [14].

According to a recently published meta-analysis the global prevalence of sarcopenia ranged from 10 to 27% in populations of older adults ≥ 60 years [15]. Further the global prevalence of SO among older adults was 11% [8]. So, sarcopenia and SO are prevalent conditions, with multiple negative health outcomes and should be given special attention [16]. Despite the large burden on patients and health care systems, the awareness of the importance of skeletal muscle maintenance in obesity is low among clinicians and scientists [3, 16].

A recent meta-analysis on publication trends revealed that despite an increase in global research on sarcopenia, the Nordic countries were only limitedly represented [6]. Nordic countries may differ from other regions on aspects associated with the prevalence and trajectory of sarcopenia and SO and challenge the representativeness of research findings from other parts of the world. These include a different prevalence pattern of noncommunicable diseases [17], different life-style behavior and life-style associated risk factors [15, 18], and higher life expectancy [18].

The Nordic countries including Sweden, Finland, Iceland, Norway, Denmark, and three autonomous areas (Åland Islands, Greenland and Faroe Islands) share common elements of social and economic policies such as a comprehensive publicly financed health care system [18, 19]. Additionally, these countries have a strong tradition of collaboration including a common vision of a socially sustainable region by promoting equal health and inclusive participation in society for older adults [20]. Therefore, more insight into the etiology, prevalence, and risk factors for sarcopenia and SO among older adults is a prerequisite for the development and implementation of effective strategies to prevent and treat these complex geriatric conditions in this geographic region. So, the aim of this study is to conduct a scoping review to systematically identify and map the available evidence while also addressing knowledge gaps and exploring the following research questions: (1) What are the prevalence of sarcopenia and SO in older adults living in the Nordic countries? (2) Which risk factors or contributing conditions are involved in the development of sarcopenia and SO in the Nordic Countries? (3) Which interventions to prevent or counteract negative health outcomes of sarcopenia and SO have been tested or implemented among older adults living in the Nordic countries?

## Methods

### Identification of relevant studies

The development and reporting of this review were done by following the Preferred Reporting Items for Systematic Reviews and Meta-analyses (PRISMA) guidelines [21].

The literature search was developed to target three main areas: Sarcopenia, sarcopenic obesity, and aging (See Appendix 1 for full search strategy). All studies published before the end of February 2023 were included in this scoping review. The optimal sensitivity of search was obtained by simultaneous search of the following databases: PubMed, Embase, and Web of science (WOS). Additionally, a detailed search for grey literature was performed in relevant databases (e.g., Research Portal Denmark, Libris, Oria, Research.fi). Besides, reference lists of the included studies were reviewed to identify eligible studies. Duplicates and non-peer reviewed evidence (e.g., PhD thesis) were excluded but if the latter contained published articles of relevance, these were included. If more than one publication on similar outcomes (e.g., prevalence) were based on a single study, just one publication was included. Data were extracted from large studies with combined data from several countries only when findings were presented separately for the Nordic countries.

### Inclusion and exclusion criteria

*The inclusion criteria were as follow*: Broad selection criteria were used to be comprehensive: (1) studies with any outcome (e.g., prevalence, risk factors, etc.) to address our research questions on sarcopenia and SO, (2) studies on subjects with age ≥ 60 years in any type of settings (e.g., community, nursing homes, general practice, hospital, outpatients, homecare, etc.), (3) studies using any definition of sarcopenia and SO without restriction for criteria and cutoff values, (4) all type of study designs (e.g., randomized control trials, cohort studies, cross-sectional, etc.), (5) studies should be conducted in the Nordic countries *The exclusion criteria are as follow*: (1) studies without relevant outcome to sarcopenia or SO, (2) studies without sufficient information to determine eligibility.

### Study selection and data extraction

Two independent researchers screened literature and conducted data extraction. Any discrepancies between them were resolved through discussion.

**Fig. 1:**
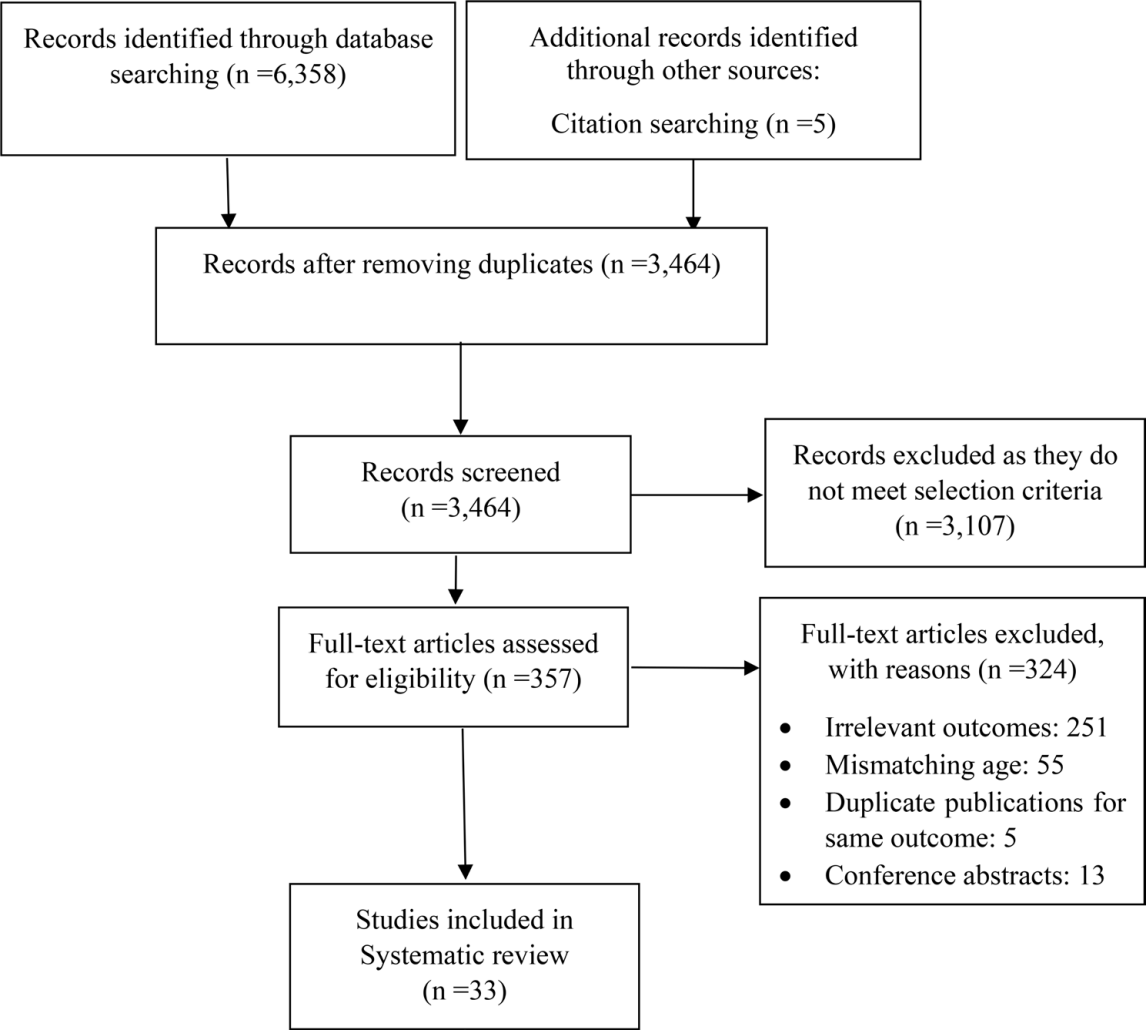
PRISMA diagram for searching resources

First, duplicates were removed by using EndNote 20.6 software, then titles and abstracts were screened to narrow down the list of potentially eligible studies. Finally, the full text review was done to examine in detail the studies that were not excluded in first step. For more clarification, the reasons for the exclusion were recorded (Fig. 1).

The following information was extracted: (1) study characteristics (e.g., first author’s name, country, year of publication), (2) characteristics of the target population (e.g., age, sex), (3) study design, setting, intervention duration and follow-up time (if applicable), measurements, tools, criteria, and results.

## Results

### Study selection

A combined total of 6,358 studies were identified through the initial electronic database and grey literature searches. An additional five articles were identified through other sources (citation searching). After removing duplication, 3,464 articles remained. A total of 3107 articles were excluded based on screening titles and abstracts. Out of the remaining 357 studies, 324 were excluded after the full-text review. Finally, 33 studies met our inclusion criteria and were included in this current scoping review [22–54] (Fig. 1).

### Study characteristics

Table 1 summarized characteristics of the included studies.

**Table 1:** Characteristics of the included studies

Study	Field	Sample Size (by sex)	Age (Range/Mean)	Design	Setting	Measurement	Tool	Criteria	Results
Sallfeldt et al., (2023), Sweden [22]	S	M: 1266	69–81	Cohort	Community dwelling	Anthropometry, BC, Muscle strength, Muscle quantity	BMI, DXA, HGS, CST, ASM	EWGSOP2	Prevalence: Baseline: 5.6% Follow-up: 12.0% Incidence proportion: 9.1%
Sääksjärvi et al. (2023), Finland [23]	S, SO	F: 711 M: 374	70–100	Cross-sectional	Community-dwelling	Anthropometry, muscle strength, probable sarcopenia, mortality, lifestyle parameters	BMI, WC, HGS	EWGSOP2 (HGS only), WHO criteria for obesity (BMI& WC cut-points)	Probable sarcopenia: 20.4% Probable SO (BMI): 5.8% Probable SO (WC, WHO): 12.6% Probabale SO (WC, alternative): 6.5% Probable SO (BMI or WC, alternative): 7.7%
Karlsson et al., (2022), Sweden [24]	S	M:257	71	Longitudinal	Community dwelling	Dietary assessment, Muscle strength, muscle power, Physical performance, Anthropometry, BC	Seven- days by a pre-coded menu book, HGS, CST, Self-chosen comfortable walking speed, DXA	EWGSOP	Prevalence: Follow-up: 19% High consumption of vegetables, green salad, fruit, poultry, rice& pasta, was associated with a lower prevalence of sarcopenia.
Dolin et al., (2022), Denmark [25]	S	F:38 M:26	65–93	Cross-sectional	Patients with colorectal cancer in outpatient clinic	Muscle strength, Physical performance, Muscle quantity	HGS, CST, DXA, ALM	EWGSOP2	Prevalence: 13%
Paajanen et al. (2022), Finland [26]	S	F: 63 M: 417	75.6	Retrospective cohort study	Patients with infrarenal abdominal aortic aneurysms	Psoas muscle area	CTA	Psoas muscle area values < 8.0 and < 5.5	Prevalence: 58.5%
Sobestiansky et al., (2021), Sweden [27]	S	F:38 M:18	84.1	Cross-sectional	Inpatients in geriatric care	BC, Muscle strength, Nutritional assessment	DXA, HGS, CST, MNA-SF	EWGSOP2	Prevalence: Sarcopenia: 46% Malnutrition: 60–64%
Papaioannou et al., (2021), Sweden [28]	S	F:122 M:69	65–70	Cross- sectional	Community dwelling	Dietary intake, Anthropometry, BC, Muscle strength, PA,	FFQ, weight, height, BIA, HGS, chair rise, accelerometry	EWGSOP2	Adherence to a healthy diet has beneficial effects on sarcopenia risk.
Wallengren et al., (2021), Sweden [29]	S	F:593 M:448	70 & 85 years old	Cross-sectional	Population Register	BC, Muscle strength, Physical performance	DXA, HGS, gait speed	EWGSOP1 EWGSOP2	Prevalence (age 70) EWGSOP1: 3.1% (F: 1.8%; M: 4.6%) EWGSOP2: 2.8% (F: 2.2%; M: 3.6%) Prevalence (age 85) EWGSOP1: 54% (F: 50; M: 61%) EWGSOP2: 54% (F:54%; M:53%)
Scott et al., (2021), Sweden [30]	S	F:1688 M:1646	70	Cohort	Community dwelling	BC, Muscle strength, Physical performance	DXA, HGS, TUG	EWGSOP2	Prevalence: Sarcopenia: 1.0% Probable sarcopenia: 0.8% Higher PA are associated with a decreased likelihood of sarcopenia.
Veen et al., (2021), Sweden [31]	S	F:122 M:71	65–70	Cross-sectional	Community dwelling	Muscle mass, SRS	BIA, HGS, 5STS, SMI	EWGSOP	Adherence to MSA is related to lower sarcopenia risk.
Mølmen et al., (2021), Norway [32]	S	F:51 M:43	68.0	RCT, (28 weeks)	Healthy participants and COPD patients	Muscle strength Performance tests, Muscle mass, Muscle quality, BC	Unilateral maximal strength and muscular performance, DXA	NP	The intervention reduced the number of participants that could be defined as sarcopenic from 16–12%.
Simonsen et al., (2021), Denmark [33]	S	F:36 M:95	65.9	Cross-sectional	Participants referred to surgery for gastrointestinal tumors	Lean soft tissue, Skeletal muscle area	DXA, CT scan	For DXA: EWGSOP2 For CT: Muscle area at the L3 level, normalized to height	Prevalence: With DXA: 11.5% With CT: 19.1%.
Faxén-Irving et al., (2021), Sweden [34]	S	F:57 M:35	86.5	Cross-sectional	Nursing-home	Nutritional assessment, BC, Muscle strength, Frailty	MNA-SF, BIA, Chair stand test, SARC-F Questionnaire, FRAIL Questionnaire	EWGSOP2 & GLIM for Malnutrition	Prevalence: Pre-frail: 51% Probable sarcopenia: 44% Sarcopenic: 29% Malnourished: 17%. All three above conditions: 7%
Björkman et al. (2020), Finland [35]	S	F:148 M:70	75–96	RCT	Community-dwelling adults with sarcopenia	Physical performance, muscle strength, BC	SPPB, HGS, BIS	Low HGS *or* slow gait speed *or* low calf skeletal muscle index	No significant differences in physical performance, muscle strength and BC after intervention
Jyväkorpi et al. (2020), Finland [36]	S	M: 126	87	Cross-sectional (Part of Helsinki Businessmen study)	Community-dwelling	BC, muscle strength, PF, dietary intake, nutritional status	HGS, SPPB, 3-day food records, Mini Nutritional Assessment	EWGSOP2	Prevalence: Probable sarcopenia: 38.1% Sarcopenia: 21.4% Inverse association between sarcopenia and total energy, protein, plant protein, fish protein, fiber, total fat and mono- & polyunsaturated fats, and vitamin D
Probert et al., (2020), Sweden [37]	S	n_1_: F:49 M:29 n_2_: F:47 M:29	81.0 80.0	2 Cohorts	Patients with hip fracture at hospital	Malnutrition, Muscle strength, Muscle mass	GLIM-criteria for malnutrition, HGS, Calf Circumference	EWGSOP2	Prevalence 2008 : Sarcopenia: 25% Malnutrition: 59% Prevalence 2018: Sarcopenia: 11% Malnutrition: 37%
Sjöblom et al., (2020), Finland [38]	S	F : 610	66–71	Part of RCT OSTPRE-FPS*	Postmenopausal community-dwelling women	BC, PF, dietary intake	DXA, HGS, Food record (3-days)	EWGSOP	Higher PA and protein intake was associated with greater PF and lower fat mass
Berens et al., (2020), Sweden [39]	SO	n_1_: F:319 M:202 n_2_: M:288	75.6 87	2 Cohorts	Community-dwelling	BC, Muscle strength	DXA, HGS, CST, BIS	EWGSOP2 combined with 3 definitions for obesity	Prevalence: 4–11% SO was associated with higher risk of dying (cohort_1_). Obesity without sarcopenia was related to better survival (cohort_2_).
Nielsen et al., (2020), Denmark [40]	S	F: 297 M: 323	65–93	Cross-sectional	Home-dwelling	BC, Muscle strength, PF, BMD	DXA, HGS, CST	EWGSOP2 & WHO guidlines for Osteoporosis	Prevalence: Osteosarcopenia 1.5%. Sarcopenia in individuals with osteoporosis: 7–8%. Osteoporosis in individuals with sarcopenia: 61.5%.
Van Ancum et al. (2020), Denmark [41]	S	F: 62 M:33	75–85	Cross-sectional (Data from the Falls outpatients’ cohort)	Older adults referred to a geriatric outpatient clinic	Muscle strength, PF, BC	HGS, gait speed, DXA	EWGSOP EWGSOP2	Prevalence: EWGSOP M: 24.2% F: 17.7% EWGSOP2 (HGS&ASM; HGS&ASM/height^2^): M: 15.2%; 15.2% F: 11.3%; 6.5%
Björkman et al., (2019), Finland [42]	S	F:194 M:68	83.0	Longitudinal	Community-dwelling	Muscle mass, Muscle strength, PF	BIS, HGS, SPPB	NP	Lower limb CRi-SMI is an independent long- term predictor of the PF for sarcopenic people.
Björkman et al., (2019), Finland [43]	S	F:194 M:68	75	Cross-sectional& Longitudinal	Community-dwelling	Muscle mass, Muscle strength, PF	BIS, HGS, SPPB	NP	Muscle mass, muscle strength and physical performance are suitable targets for the prevention of sarcopenia-related over-mortality.
Olesen et al., (2019), Denmark [44]	S	F:98 M:84	57.4	Cohort	Pancreatitis outpatients at center for Pancreatic Diseases	BC, Muscle mass, Muscle strength, Muscle function, PF, Quality of life	BIA, HGS, TUG, QLQ-C30 questionnaire	EWGSOP	Prevalence: 17.0%. Sarcopenia was associated with reduced quality of life, PF and increased risk of hospitalization. EPI was an independent risk factor for sarcopenia.
Sobestiansky et al., (2019), Sweden [45]	S	M:287	85–89	Longitudinal	Community-dwelling	BC, Muscle strength, Physical performance	DXA, HGS, GS, CST	EWGSOP EWGSOP2 FNIH	Prevalence: Probable sarcopenia (EWGSOP2): 73% Sarcopenia: EWGSOP: 21% EWGSOP2: 20% FNIH: 8% Reduced muscle strength was a major determinant of sarcopenia.
Vikberg et al. (2019), Sweden [46]	S	F:54 M:38	70.9	RCT (10 Weekes)	Community-dwelling with pre-sarcopenia	Functional strength, physical function, muscle strength, LBM	SPPB, HGS, DXA	EWGSOP	Significant improvement of SPPB scores in males. LBM increased and FM decreased in the total intervention group.
Isanejad et al. (2018), Finland [47]	S	F: 554	≥ 65	Cross-sectional and longitudinal (Part of RCT OSTPRE-FPS*)	Community-dwelling postmenopausal women	Muscle strength, PF, BC, Adherence to Mediterranean diet and Baltic Sea Diet	HGS, SPPB, DXA, 3-day food record	EWGSOP	Prevalence 24.2% Participants with lower adherence to Mediterranean and Baltic Sea Diets lost more total body LM and SMI.
Mikkola et al., (2018), Finland [48]	SO	F: 603 M:473	61.2	Part of cohort HBCS**	Community-dwelling	BC, WC, Physical performance	BIA, Senior fitness test	NP	Body composition measures that reflect adiposity predict physical performance better than measures that reflect lean mass.
Ottestad et al., (2018), Norway [49]	S	F: 218 M:199	74	Cross-sectional	Home-dwelling	Amino acids concentrations in plasma, Protein intake, SM	NMR spectroscopy, 24-h dietary recalls, BIA	EWGSOP	Prevalence All: 22% F: 32% M:11% People with sarcopenia have a lower absolute intake of protein and lower non-fasting plasma concentrations of leucine and isoleucine compared with non-sarcopenic subjects.
Steihaug et al., (2017), Norway [50]	S	F:152 M:50	≥ 65	Cross-sectional	Patient with hip fracture at hospital	Muscle strength, Total body mass	HGS, Anthropometry (Heymsfield method)	EWGSOP	Prevalence: 37% Sarcopenia was positively associated with age, polypharmacy, and negatively associated with BMI and albumin.
Jacobsen et al., (2016), Norway [51]	S	F: 76 M:44	82.6	Cross-sectional	Hospital (Acute patients)	Nutritional assessment, PF, Muscle strength	MNA-SF, SPPB, HGS, GS	EWGSOP	Prevalence: Sarcopenia: 30% Malnutrition or at risk: 75% Sarcopenia is associated with a decline in nutritional status.
Jansen et al., (2015), Denmark [52]	S	F: 9 M:40	61.0	Cross-sectional case control	Diabetic patients with acute or chronic Charcot osteoarthropathy	Fat mass, Muscle strength	DXA, ALM	ALM relative to height squared	Prevalence: 9.1–40.0% Compared to the reference values, the studied population with diabetes had higher rates of obesity and sarcopenia.
Frost et al. (2014), Denmark [53]	S	M: 593	60–74	Observational	Population-based	BC	DXA, LEP	EWGSOP, (By using LEP)	Prevalence: 4.8% and 8.5% based LEP or LELB T-score, respectively.
Patil et al., (2013), Finland [54]	S	F: 409	70–80	Cohort	Community-dwelling	Muscle mass, Muscle strength, Muscle performance, BC	SMI, HGS, GS, DXA	EWGSOP IWG & WHO guidlines for Osteopenia	Prevalence: Sarcopenia: EWGSOP: 0.9% IWG: 2.7% Osteopenia: 36%

S: Sarcopenia; F: Females; M: Males; BC: Body composition; BMI: Body mass index; DXA: Dual energy X-ray absorptiometry; HGS: Hand grip strength; CST: Chair stands test; CTA: Computed Tomography Angiogram; ASM: Appendicular skeletal muscle mass; EWGSOP: European Working Group on Sarcopenia in Older People; EWGSOP2: Revised consensus from the European Working Group on Sarcopenia in Older People; ALM: Appendicular lean mass; NP: Not provided; MNA-SF: Mini nutritional assessment-short form; FFQ: Food frequency questionnaire; PA: Physical activity; SMI: Skeletal muscle mass index; BIA: Bioelectrical impedance analyses; TUG: Timed Up and Go; MSA: Muscle-strengthening activities; 5STS: Five times sit-to-stand time; SRS: Sarcopenia risk score; RCT: Randomized control trial; COPD: Chronic obstructive pulmonary disease; GLIM: Global Leadership of Malnutrition; PF: Physical function; SO: Sarcopenic obesity; BMD: Bone mineral density; BIS: Bioimpedance spectroscopy; SPPB: Short physical performance battery; CRi-SMI: Calf intracellular resistance skeletal muscle index; TUG: Timed up-and-go test; EPI: Exocrine pancreatic insufficiency; FNIH: National Institutes of Health Sarcopenia Project; GS: Gait speed; ULSAM: Uppsala Longitudinal Study of Adult Men; WC: waist circumference; SM: Skeletal muscle mass; T: Total; LBM: Lean body mass; IWG: International Working Group on Sarcopenia; LEP: leg extension power; LELB: Lower extremity lean mass

* OSTPRE-FPS: Osteoporosis Risk Factor and Prevention – Fracture Prevention Study; **HBCS: Helsinki Birth Cohort Study

The number of documents showed an increasing trend between 2020 and 2021. A peak in the number of publications was observed in 2021 (24.2% of all documents). All the studies were conducted across four (Denmark, Norway, Sweden, and Finland) out of the five Nordic countries and three autonomous areas. The highest contribution in this field was made by Sweden (*n* = 12).

Most studies were conducted in community-dwelling settings [22–24, 28, 30, 31, 35, 36, 38–40, 42, 45–49, 54]. Seven studies included patients with acute diseases (hospital-setting) [26, 27, 33, 37, 50–52], while four studies included patients with chronic conditions (out-patient setting) [25, 32, 41, 44], and one study including nursing-home residents [34]. In terms of study design, most of the studies were observation studies with a cross-sectional or longitudinal design (22–31, 33, 34, 36–39–45, 46–54), while three studies [32, 35, 46] applied interventions. It appears, however, that one study [32] out of the above three interventions is sub-project conducted within the framework of larger intervention program. Sample size ranged from 49 in a cross-sectional case control study [52] to 3334 in a cohort study [30].

Five studies were among males only [22, 24, 36, 45, 53] and three studies included females only [38, 47, 54]. The rest of the studies had a mixed sample. Top subject area was sarcopenia (31 out of the 33 included studies), and on this subject, publications were categorized into the following research areas (with some studies addressing more areas): prevalence [22–27, 29, 30, 33, 35, 36, 37, 40, 42, 44, 45, 47, 49–51, 52–54], risk factors [24, 27, 28, 30, 31, 34, 38, 40, 42, 44, 47, 49–51], and effectiveness of interventions on sarcopenia or indicator of sarcopenia [32, 35, 46].

In most studies sarcopenia was defined according to the criteria set by the European Working Group on Sarcopenia in Older People in the updated version from 2019 (EWGSOP2) (*n* = 15) or the original version from 2010 (EWGSOP) (*n* = 14). However, in some studies multiple criteria such as EWGSOP, EWGSOP2, and National Institutes of Health Sarcopenia Project definition (FNIH) were applied [27, 39, 43], and in other studies alternative criteria were used [26, 33, 35, 45, 57].

Different assessment methods of muscle mass including Dual energy X-ray absorptiometry (DXA) [22, 24, 25, 27, 29, 30, 32, 33, 38–41, 45–47, 52–54], Bioelectrical Impedance Analysis (BIA) [28, 31, 34, 44, 48, 49], Bioimpedance Spectroscopy (BIS) [35, 42, 43], Computed Tomography (CT) [33], and Computed Tomography Angiogram (CTA) [26] were used in the included studies.

SO were defined by the co-existence of sarcopenia with obesity. Studies on SO used the EWGSOP2 criteria [39], or the EWGSOP2 criteria for hand grip strength only (probable sarcopenia) [23] in combination with obesity estimated from BMI cut points [23, 39], waist circumference [23, 39], and fat mass percentage [39]. Lastly, one study used measures of body composition measures that reflect adiposity as estimates of SO [48].

Four studies reported the prevalence of “probable sarcopenia” [23, 30, 36, 45], while two studies reported the prevalence of sarcopenia and comorbidities (e.g., osteopenia, pre-frailty, malnutrition) [33, 40].

### Narrative synthesis

Due to the heterogeneity of the studies in definition of sarcopenia, settings, and sample size, the overall reported prevalence was variable and ranged from 0.9% [54] to 58.5% [26]. However, according to the most commonly used criteria (EWGSOP2) the highest (46%) and lowest (1%) prevalence of sarcopenia was reported in Sweden among inpatients in geriatric care [27], and community-dwelling older adults [30], respectively.

Prevalence of sarcopenia according to population and definition criteria is illustrated in Table 2. Higher prevalence rates of sarcopenia were found in females compared to males among community-dwelling older adults [49] and in older adults acutely admitted to hospital [51]. Further, acutely admitted female patients also presented with more severe sarcopenia compared to male patients [51].

**Table 2:** Prevalence of sarcopenia according to population and definition criteria

Population \ Definition criteria	EWGSOP2	EWGSOP
Community-dwelling	1.0-21.4% [20, 27, 28, 34, 38, 43]	0.9%*-54% [22, 27, 43, 45, 47, 52]
Patients in-hospital	11-46% [25, 35]	30% [48, 49]
Out-patients	11.3*%-15.2**% [23, 39]	17.7%**-24.2*% [39, 42]
Nursing home	29% [32]	-------

*Females only

**Males only

Frequency of sarcopenia was higher (9.1–40.0%) in patients with diabetes (with and without complications of charcot osteoarthropathy), compared to age-matched healthy adults [52].

The prevalence of “probable sarcopenia” ranged between 20.4% (reduced muscle strength only) and 38.1% (fulfilling one of the following criteria: reduced muscle strength, reduced muscle mass, or low physical function) in Finnish community-dwelling adults [23, 36], while longitudinal studies on Swedish community-dwelling old (70 years) and very old adults (≥ 85 years) the prevalence of “probable sarcopenia” (reduced muscle strength only) ranged from 1.8 to 73%, respectively [30, 45]. Lastly, in a Swedish study among nursing home residents the prevalence of probable sarcopenia was 44% (evaluated by an impaired chair stand test) [34].

Prevalence of Osteosarcopenia (sarcopenia and osteoporosis) was 1.5% [36], and the prevalence of co-occurrence of all three following conditions: pre-frail, malnutrition, and sarcopenia was 7% [34].

We only identified two studies with prevalence of SO [39] and probable SO [23]. The prevalence of SO in a Swedish population was 4% and 11% in females and males, respectively, while the prevalence of probable SO among Finnish community-dwelling ranged between 5.8% and 12.6%, depending on the criteria to define the obesity (e.g., BMI, waist circumference, etc.) [23].

Several studies investigated aspects of etiology and risk factors for sarcopenia [24, 27, 28, 30, 31, 34, 36, 38, 40, 42–44, 47, 49–51] and one study focused on SO [49]. Higher physical activity was associated with a decreased likelihood of sarcopenia [30]. In addition, adhering to world health organization (WHO) guidlines for physical activity and the Nordic nutritional recommendations for protein intake was positively associated with greater physical function and lower fat mass in older female community-dwellers [38]. In older adults who are physically active, eating a healthy diet (based on the frequency of intake of favorable food like fish, fruits, vegetables, and whole grains versus unfavorable foods like red/processed meats, desserts/sweets/sugar-sweetened beverages, and fried potatoes) was associated with lower risk of sarcopenia [28]. Further, among older adults who already meet the physical activity guidelines, additional engagement in muscle-strengthening activities was associated with a lower sarcopenia risk score and improved muscle mass and chair rise time [31].

Associations between sarcopenia, risk of sarcopenia and malnutrition or nutritional status was identified in geriatric patients [27, 51], older patients with hip fracture [50], nursing home residents [34] and in community-dwelling older adults [49]. Moreover, the importance of nutritional intake was investigated in the following studies [24, 36, 47]. A study among community-dwelling men revealed an inverse association between total energy intake, protein intake (total, plant, and fish protein), intake of dietary fibers, fat (total and unsaturated), and vitamin D with sarcopenia status [36]. In a cohort of 71-year-old men a dietary pattern characterized by high consumption of fruit, vegetables, poultry, rice and pasta was associated with lower prevalence of sarcopenia after 16 years [24]. A longitudinal Finnish study on sarcopenia indices among postmenopausal older women, showed that lower adherence to the Mediterranean (focuses on high consumption of olive oil) or Baltic Sea (focuses on the dietary fat quality and low-fat milk intake) diets resulted in higher loss of lean mass over a 3-year period [47]. Further, a higher adherence to the Baltic Sea diet was associated with greater lean mass and better physical function, and higher adherence to the Mediterranean diet was associated with greater muscle quality [47].

In a study of patients with hip fracture age, polypharmacy, and low albumin levels was associated with sarcopenia [50]. Exocrine pancreatic insufficiency was an independent risk factor for sarcopenia [44]. This study also revealed that sarcopenia was associated with reduced quality of life, physical function, and increased risk of hospitalization [44]. In a longitudinal study of community-dwelling adults (+ 75 years) at risk of sarcopenia, high physical function, muscle strength, muscle mass and low BMI predicted better physical function and reduced need for care after four years [42]. Furthermore, in community-dwelling adults with sarcopenia, muscle mass, muscle strength and physical function are independent predictors of all-cause mortality. As a result, they have been proposed by researchers as targets for the prevention of sarcopenia-related over-mortality [43]. Lastly, community-dwelling older adults with sarcopenia had lower bone mineral density compared to those without sarcopenia and they were more likely to develop osteoporosis (Osteosarcopenia) [40].

Regarding SO risk factors, a longitudinal study among community-dwelling older adults in Finland found that SO (operationalized by measures of adiposity) were associated with poorer physical function after ten years [48].

Our literature search identified three randomized controlled trials investigating the effectiveness of interventions to prevent or counteract sarcopenia in older adults of Norway, Finland, and Sweden, respectively [32, 35, 46]. The Norwegian study [32] was a double-blinded randomized controlled trial (RCT). The study included those who were at risk of developing sarcopenia, including patients with chronic obstructive pulmonary disease (COPD) or individuals who showed diagnostic indications of sarcopenia. Participants received either vitamin D_3_ or placebo supplementation for 28 weeks. Additionally, resistance training sessions were provided to all participants from weeks 14 to 27. Vitamin D supplementation did not significantly affect response to resistance training in older adults at risk of sarcopenia with or without COPD [32].

Furthermore, a RCT among pre-sarcopenic Swedish older adults investigated the effectiveness of three weekly sessions of instructor-led progressive resistance training in combination with a non-mandatory daily nutritional supplement (175 kcal, 19 g protein) compared to control group. The 10 weeks intervention resulted in significant between group improvements of physical function and a significant improvement in body composition in the intervention group [46].

Another intervention study revealed that a 12-month intervention with two daily nutritional supplements (each containing 20 g whey protein) did not attenuate the deterioration of physical function and muscle mass in sarcopenic older community-dwelling adults compared to isocaloric placebo supplements or no supplementation. All participants were given instructions on home-based exercises, importance of dietary protein and vitamin D supplementation [35].

## Discussion

Based on our broad literature search 33 studies were identified that concerned sarcopenia and SO and met the inclusion criteria. However, research on SO was very limited with only three studies identified. Narrative synthesis of the included studies revealed that the most reported classification tool for sarcopenia in Nordic countries was the EWGSOP2. Moreover, some studies estimated sarcopenia using EWGSOP. The overall prevalence of sarcopenia in Nordic countries according to EWGSOP2 ranged between 1% and 46% [25, 28]. The prevalence of SO, however, was reported only in one study in Sweden (4–11%) [39]. Even though the previous systematic reviews and meta-analysis have reported the prevalence of sarcopenia and SO in different regions and settings (e.g., community-dwelling, nursing home, etc.) [8, 15, 55, 56], this current scoping review is to the best of our knowledge the first study that provides an overview of research on sarcopenia and SO in the Nordic countries.

Based on our findings from 24 studies, there were large variability in prevalence of sarcopenia in studies conducted in the Nordic countries. We think that the wide variation in estimated prevalence of sarcopenia in our scoping review might be due to a different definition/diagnostic criterion (e.g., EWGSOP, EWGSOP2, FNIH), methodology to measure muscle mass (DXA, BIA, CT), and heterogeneity in characteristics of the study population (e.g., setting, age, medical conditions, co-occurrence of multiple risk factors). A previous study on prevalence of sarcopenia in Swedish older people showed significant differences between prevalence of sarcopenia based on EWGSOP2 and EWGSOP1 [29]. Therefore, researchers stressed that prevalence is more dependent on cut-offs than on the operational definition [29, 57]. Further, we know that various international sarcopenia working groups have issued expert consensus and such diagnostic criteria are being updated [4, 58]. Since the revision of criteria focuses primarily on the adjustment of cut-off values, the main reason for differences in prevalence even when using an updated version of one diagnosis criteria is modification in cut-off values. For instance, if the cut-off value for gait speed was increased by 0.2 m/s, the prevalence of sarcopenia may increase by 8.5% [57]. Meaning that even a small change in cut-off value can have a big impact on how sarcopenia is diagnosed. Besides when we take definition criteria into account (Table 2), the prevalence of sarcopenia is still variable in the population of community-dwelling adults for instance. We believe it is basically because studies have applied different assessment tools and tests to identify older adults with low muscle mass and muscle strength, although using the same definition criteria (Table 1). Previous studies have illustrated that choice of methodology to assess muscle strength (e.g., hand grip strength, chair rise) [59] and muscle mass (e.g., DXA, BIA, anthropometry) [60–62] in older adults may impact findings and this variability may explain some of the variability in our findings. So, adherence to the latest uniform diagnostic criteria for future studies is recommended to simplify the comparison of findings within the same country, across countries, and regions. Moreover, we suggest that medical community particularly GPs to come to an agreement on assessment methods for muscle mass and muscle strength and the use of one set of definition criteria for sarcopenia.

In previous meta-analyses [15], sub-group analyses based on region and classification tool, revealed that the prevalence of sarcopenia was higher in European studies using EWGSOP (12%) compared to rest of the studies using Asian Working Group for Sarcopenia (AWGS), FNIH, and EWGSOP (3%) [15]. In our scoping review, we also found a high prevalence of sarcopenia in Nordic countries. Longevity and life expectancy is higher in the Nordic countries compared to estimates for rest of the world [18], which means that in this region many people reach old age, and consequently they are more likely to be diagnosed with sarcopenia as an age-related disorder. Therefore, the authors of this current scoping review emphasis the importance of preventive strategies targeted major risk factors and effective interventions to limit the consequences of sarcopenia in the Nordic populations. Besides, we think that the health care system in the Nordic countries should be better equipped with the necessary healthcare resources for both a timely diagnosis and dealing with this major age-related issue in the years to come. However, due to the limitations regarding the timely diagnosis, we highly recommend a comprehensive approach including establishment of support services, implement educational programs, offer training for health care professionals, and engage the community.

Many countries have conducted research on SO [7, 39, 63–65]. Based on our findings, however, among the Nordic countries only Sweden and Finland have investigated the prevalence of probable SO and SO [23, 29]. Besides, we only found one study investigating the association between body adiposity and physical function over time [54]. We did not find any literature on risk factors or interventions among older adults with SO in this region. Therefore, we call on medical and research community in Nordic countries to attach importance to screening of SO in elderly people to capture a full picture of this public health risk to aging society and allocate healthcare resources accordingly.

In terms of risk factors for sarcopenia, our study revealed that malnutrition, low levels of physical activity, specific diseases (e.g., diabetes, osteoporosis), inflammation, polypharmacy (multiple medicines), BMI, and ageing are potential risk factor for sarcopenia in populations of the Nordic region. However, evidence on risk factors derived mainly from cross-sectional associations [27, 28, 30, 31, 34, 40, 44, 49–51], and only to a limited extend from longitudinal studies [24, 38, 43, 47]. Therefore, the associations between risk factors and sarcopenia should be interpreted with caution due to the possibility of reverse causality and confounding affecting the results. Moreover, our findings on risk factors mainly came from community-dwelling older adults, and only to a limited extend hospital and nursing home settings. We think that risk factors may vary depending on population characteristics (e.g., age, sex, health condition) and setting (e.g., hospital, nursing home, community). Therefore, we encourage researchers of the Nordic countries to perform well-designed prospective cohort studies in different settings to enhance the possibility to establish causal inference as well as understanding degree and direction of changes over time.

A recently published meta-analyses revealed a higher risk of having polypharmacy in Europe among individuals with sarcopenia compared to people without this condition [66]. A nationwide register-based study in Swedish population also showed that the prevalence of polypharmacy has increased in Sweden over the last decade [67]. Sarcopenia itself is associated with morbidity (identified by specific disease or inflammatory markers) and different health-related outcomes (e.g., disability) [7]; therefore, future research should investigate whether polypharmacy is a major factor to sarcopenia development [66]. Although we lack information on polypharmacy in Nordic countries other than Sweden, we encourage researchers in this region to examine the above research gap in their future studies.

According to previous studies physiological changes in ageing include systemic low-grade inflammation which results in insulin resistance, affect protein metabolism and leads to increased muscle wasting [68]. Acute and chronic disease may increase the inflammatory response and accelerate age-related loss of muscle mass and increase risk of sarcopenia [68, 69]. Hence, we think that special attention should be made by health care professionals particularly GPs to older adults with acute or chronic conditions to limit the risk of sarcopenia.

Literature from the Nordic countries also indicated that higher levels of physical activity and different dietary patterns (e.g., higher protein intake, fruit, vegetables, fibers) were associated with reduced risk of sarcopenia or improvement in indicators of sarcopenia. There was a large heterogeneity in the studied aspect which makes direct comparison of studies difficult. Nevertheless, according to findings from a recent systematic review of meta-analyses on sarcopenia the identified risk factors are in alignment with previously identified risk factors globally [70]. Other potential lifestyle-related risk factors suggested from the above meta-analysis included smoking and extreme sleep duration. However, we did not identify studies investigating these health behaviors in the Nordic populations. Therefore, high-quality cohort studies are needed to deeply understand such associations with the risk of sarcopenia.

In this current review, we only found three intervention studies in Nordic countries. However, two of them were sub-projects of big intervention programs, meaning that such studies were not designed explicitly for the prevention/treatment of sarcopenia. Therefore, explicit intervention studies on sarcopenia in this region is recommended.

We believe that on a global level, research on sarcopenia will carry on with nutrition, exercise, and understanding of molecular mechanisms. Furthermore, examining the link between sarcopenia and other medical conditions/diseases would be the next step [6]. In the Nordic countries, however, already performed studies have a basic and descriptive design, so that, well-designed research and advanced analyses are lacking. Hence, we recommend conducting large well-designed and adequately powered studies to (a) explore the scale of this age-related health issue on country and regional level, (b) investigate the patterns of physical activity and sedentary behavior to understand if this should be a target in older adults with SO and sarcopenia, (c) determine whether elderly populations are suffering from nutritional deficiency or are at risk of malnutrition. The latest can support further studies to assess the impact of combined physical activity and dietary intake, which are still lacking globally [6].

A previous systematic review on therapeutic strategies for SO revealed that exercise-based interventions (e.g., resistance training) reduced total adiposity and consequently improved body composition. However, evidence of other therapeutic strategies (e.g., nutritional supplementation) was limited due to scarcity of data and lack of unique definition for SO [69]. Therefore, authors suggested that more research should be done to clarify optimal treatment options for various age-groups and not only for older adults [14].

In our scoping review, the included studies, did not provide a status of either SO or the prevention/treatment methods in this region. We believe that SO is practically neglected in clinical practice and research as well, and this is mainly because it is difficult to separate it from general obesity. The consequence of lacking knowledge in this research area is that when older adults with SO are recommended weight loss- a frequently used strategy for management of general obesity- this may accelerate the loss of muscle mass and increase the severity of the sarcopenia [3]. Consequently, we think that this issue may have adverse effects both on patients (e.g., decreasing quality of their life) and on the health care system (e.g., increasing the health care demands) of this region. Therefore, we encourage researchers to perform cohort studies to understand the epidemiology and etiological basis of SO, which are poorly understood even on a global scale [8]. We think that the consensus definition on SO from the European Society for Clinical Nutrition and Metabolism (ESPEN) and European Association for the Study of Obesity (EASO) which was published in 2022 [3], can positively affect the ability to define studies on prevalence and prevention of SO. Besides, we recommend conducting further research to find the optimal treatment for SO and reduce its adverse consequences both at individual and society levels. Additionally, we think that the concepts of sarcopenia and SO might be somehow unfamiliar to health care personnel. Therefore, it is highly recommended that more information be provided to bring their attention to the significance of prevention, timely diagnosis, and treatment of these two aging disorders.

### Strengths and limitations of the study

This is the first study providing an overview of available evidence on sarcopenia and SO among older adults in the Nordic countries. These countries have important similarities in welfare sectors and on a population level and we believe that our findings will be a significant benefit for researchers and health care providers to understand the knowledge gaps and plan for future studies in this geographical region. However, the current scoping review has limitations. This review was limited to studies among individuals more than 60 years old which may limit the overview of available research in this field, as well as understanding risk factors, confounders for prevention, and the potential for early detection of these two diseases in younger age population. The included cross-sectional studies in our review cannot provide information on causality of the associations.

## Conclusion

Sarcopenia and SO are generally prevalent syndromes among older adults in Nordic countries, even though the prevalence of them varies according to the criteria for definition, population, and setting. Research among older adults with SO was very limited in this region. Besides, studies on risk factors were primarily cross-sectional and only few intervention studies were identified. Therefore, we encourage researchers performing well-designed studies (e.g., prospective cohorts) to understand the epidemiology and etiological basis of these two age-related disorders. For the next step, implementation of interventions targeting risk factors (e.g., combined physical activity and dietary intake) and evaluating of their impact on prevention or treatment of sarcopenia and SO is recommended. Furthermore, for the comprehensive advancement of muscle health in older adults, we recommend implementing interventions directed at health care personnel and encouraging more collaboration among clinicians, professional societies, researchers, and policy makers to ensure comprehensive and effective approach to health care initiatives.

### Electronic supplementary material

Below is the link to the electronic supplementary material.

Supplementary Material 1

## Data Availability

The datasets used and/or analysed during the current study available from the corresponding author on reasonable request.

## Abbreviations

SO: sarcopenic obesity
WOS: Web of science
PRISMA: Preferred Reporting Items for Systematic Reviews and Meta-analyses
EWGSOP2: European Working Group on Sarcopenia in Older People in the updated version from 2019
FNIH: National Institutes of Health Sarcopenia Project definition
DXA: Dual energy X-ray absorptiometry
BIA: Bioelectrical Impedance Analysis
BIS: Bioimpedance Spectroscopy
CT: Computed Tomography
CTA: Computed Tomography Angiogram
WHO: World Health Organization
GP: General Practitioner
RCT: Randomized Controlled Trial
COPD: Chronic Obstructive Pulmonary Disease
EASO: European Association for the Study of Obesity
