# Supplementary material for: Sarcopenia and sarcopenic obesity among older adults in the nordic countries: a scoping review
Source: BMC Geriatr. 2024 May 13;24:421. doi: 10.1186/s12877-024-04970-x (PMC11092005; doi:10.1186/s12877-024-04970-x)
Supplement: Supplementary file 1 — Supplementary Material 1 [file 12877_2024_4970_MOESM1_ESM.pdf]

Appendix 1: Search strategy for scoping review on sarcopenia and sarcopenic obesity in the Nordic countries

Database	Search syntax
PubMed	(("old population*" OR senior* OR "aged"[MeSH Terms] OR "aged"[Title/Abstract] OR "elderl*"[Title/Abstract] OR "old people"[Title/Abstract] OR "over 65"[Title/Abstract] OR "old person*"[Title/Abstract]) AND (obes* OR "overweight"[MeSH Terms] OR overweight* OR adipos* OR "adiposity"[MeSH Terms] OR "excessive weight" OR "obesity paradox" OR "sarcopenic obesit*")) AND (("sarcopenia"[MeSH Terms] OR "sarcopeni*"[Title/Abstract] OR "muscle atroph*"[Title/Abstract] OR "muscle wast*"[Title/Abstract] OR "muscle weak*"[Title/Abstract] OR "muscle los*"[Title/Abstract]))<br>**Results: 2024**
Embase	1) Old population*.mp.<br>2) Aged/<br>3) elderl*.mp. or very elderly/<br>4) old people.mp.<br>5) over 65.mp.<br>6) old person*.mp.<br>7) 1 or 2 or 3 or 4 or 6<br>8) obesity/ or obes*.mp.<br>9) obesity/ or overweight*.mp.<br>10) excessive weight.mp.<br>11) obesity paradox.mp.<br>12) 8 or 9 or 10 or 11<br>13) muscle atrophy/ or muscle atrophy*.mp.<br>14) geriatric disorder/<br>15) sarcopeni*.mp.<br>16) sarcopenia/<br>17) muscle wast*.mp.<br>18) muscle weakness/ or muscle weak*.mp.<br>19) muscle los*.mp.<br>20) 13 or 14 or 15 or 16 or 17 or 18 or 19<br>21) 7 and 12 and 20<br>**Results: 2437**
ISI/WOS	((ALL= ("old population*" OR senior* OR "aged" OR "elderl*" OR "old people" OR "over 65" OR "old person*")) AND ALL=((obes* OR "overweight*" OR adipos* OR "excessive weight" OR "obesity paradox"))) AND ALL=(("sarcopeni*" OR "muscle atroph*" OR "muscle wast*" OR "muscle weak*" OR "muscle los*"))<br>**Results: 1897**
